# Nerve Growth Factor (NGF)—Receptor Survival Axis in Head and Neck Squamous Cell Carcinoma

**DOI:** 10.3390/ijms19061771

**Published:** 2018-06-14

**Authors:** József Dudás, Wolfgang Dietl, Angela Romani, Susanne Reinold, Rudolf Glueckert, Anneliese Schrott-Fischer, Daniel Dejaco, Lejo Johnson Chacko, Raphaela Tuertscher, Volker Hans Schartinger, Herbert Riechelmann

**Affiliations:** Department of Otorhinolaryngology, Medical University of Innsbruck, Anichstrasse 35, A-6020 Innsbruck, Austria; wolfgang.dietl@student.i-med.ac.at (W.D.); angela.romani@i-med.ac.at (A.R.); susanne.reinold@i-med.ac.at (S.R.); rudolf.glueckert@i-med.ac.at (R.G.); annelies.schrott@i-med.ac.at (A.S.-F.); daniel.dejaco@i-med.ac.at (D.D.); lejo.johnson@student.i-med.ac.at (L.J.C.); raphaela111@gmx.net (R.T.); volker.schartinger@i-med.ac.at (V.H.S.); herbert.riechelmann@i-med.ac.at (H.R.)

**Keywords:** cytokeratin, necrosis, cell cycle arrest, TissueFaxs

## Abstract

Neurotrophins and their receptors might regulate cell survival in head and neck squamous cell carcinoma (HNSCC). mRNA expression of nerve growth factor (NGF) and protein synthesis of high (NTRK1) and low affinity neurotrophin (p75 neurotrophin receptor; NTR) receptors were investigated in normal oral mucosa and in HNSCC. HNSCC cell lines were treated with mitomycin C (MMC) and cell survival was investigated. Normal and malignant epithelial cells expressed NGF mRNA. NTRK1 was upregulated in 80% of HNSCC tissue, and 50% of HNSCC samples were p75NTR positive. Interestingly, in HNSCC tissue: NTRK1 and p75NTR immunohistochemical reactions were mutually exclusive. Detroit 562 cell line contained only p75NTR, UPCI-SCC090 cells synthesized NTRK1 but not p75NTR and SCC-25 culture had p75NTR and NTRK1 in different cells. NGF (100 ng/mL) significantly improved (1.4-fold) the survival of cultured UPCI-SCC090 cells after MMC-induced cell cycle arrest, while Detroit 562 cells with high levels of p75NTR did not even get arrested by single short MMC treatment. p75NTR in HNSCC might be related with NGF-independent therapy resistance, while NTRK1 might transduce a survival signal of NGF and contribute in this way to improved tumor cell survival after cell cycle arrest.

## 1. Introduction

Neurotrophins are growth factors that play important roles in physiology of organ functions but also in pathologies such as major depressive disorder, neuropathic pain, heart failure, irritable bowel syndrome, autism or mental retardation. In children in the autistic spectrum and in those with mental retardation without autism, elevated levels of the neurotrophin brain-derived neurotrophic factor (BDNF) were measured [1]. The same neurotrophin, BDNF, showed abnormally low serum levels in major depressive disorder [2]. In another pathophysiological condition, in irritable bowel syndrome, BDNF also makes a significant contribution, since increased expression of BDNF in colonic mucosa, contributes to the visceral hyperalgesia [3]. The main function of neurotrophins is the support of neuron survival, which is exemplified by the myocardial produced nerve growth factor (NGF) that is involved in the maintenance of sympathetic neuronal survival [4]. A further very important function is the interaction of the endocannabinoid system and the neurotrophins BDNF and NGF in the control of neuropathic pain mechanisms [5]. Neurotrophins either bind to low affinity (Low-affinity Nerve Growth Factor (LNGFR)/p75 neurotrophin receptor (p75NTR)) receptor [6,7] or to the high affinity Trk (tropomyosin-related kinase) tyrosine kinase receptors. The Trk family (neurotrophin receptor tyrosine kinase, NTRK) of receptors include NTRK1 (NTRK1) specific for NGF [8], NTRK2 (TrkB) specific for BDNF and neurotrophin-4 (NT-4) [9], and NTRK3 (TrkC) which is also activated by NT-4 [10]. The knowledge on neurotrophins and neurotrophin receptors in head and neck squamous cell carcinoma (HNSCC) is scarce. In HNSCC, neurotrophins play a role in perineural growth, which is a unique route of tumor progression associated with poor prognosis [11]. Neurotrophins may serve as molecular factors involved in the communication between tumor and neuronal cells. Deriving from neuronal sources, they support survival, anti-apoptosis [12] and cell-detachment inducing migratory [12] effects. We [13,14,15] and others [16] previously suggested that BDNF stimulation of HNSCC cell lines upregulated migration and invasion. Furthermore, overexpression of NTRK2 in HNSCC tumor cells was related with epithelial-to-mesenchymal transition (EMT). In contrast, less is known about NTRK1 and its ligand NGF in HNSCC, which were reported as important survival factors for oral mucosa epithelial cells [17]. Recently Yamaguchi et al. [18] published that p75NTR^+^ tumor cells are mitotically quiescent cancer stem cells. At the same time, p75NTR^+^ chemoresistant stem cells [18,19] shared some characteristics with epithelial–to–mesenchymal transdifferentiated cells [18]. In human adult oral mucosa lamina propria, p75NTR^+^ stem cells are self-renewing cells [20]. NGF growth factor and its low affinity receptor p75NTR might be involved in pleiotropic, stem-cell-like effects as self-renewal and epithelia regeneration [21]. Accordingly, p75NTR has been published in undifferentiated cell populations of oral leukoplakia as well as in oral squamous cell carcinoma (OSCC), where it was associated with poor prognosis [22]. In fact, p75NTR is supposed to play a pro-tumoral role by contributing to drug resistance [23]. Based on intracellular adaptor molecule availability, and post-translational modifications, p75NTR might be involved in cell-fate decisions just by taking part in multiple signaling pathways [21]. 

HNSCC is among the seven most common malignancies worldwide. The development of HNSCC is related to tobacco use and alcohol consumption, as the most identified risk factors, but human papilloma virus (HPV), a sexually transmitted infection, has increasing significance as another primary cause of HNSCC [24]. HPV-positive tumors not only have a different etiology, but they also remarkably better respond to radio(chemo)therapy [25], as well as surgery [26], compared to HPV-negative tumors. Concluding, the HPV-positive patients have a better prognosis. In this work, we investigated the hypothesis that NGF and its high or low affinity receptors might elaborate a survival signaling pathway in HNSCC. Based on the clear etiological and therapy response differences in HPV^+^ and HPV^−^ HNSCC patients, we also compared the survival relation of NGF and its receptors in this two HNSCC patient groups and in related cell lines.

## 2. Results

### 2.1. Gene Expression of NGF and Protein Synthesis of NTRK1 and p75NTR in Oral Mucosa, HNSCC Tumor Tissue and in HNSCC Tumor Cell Lines

**Figure 1:**
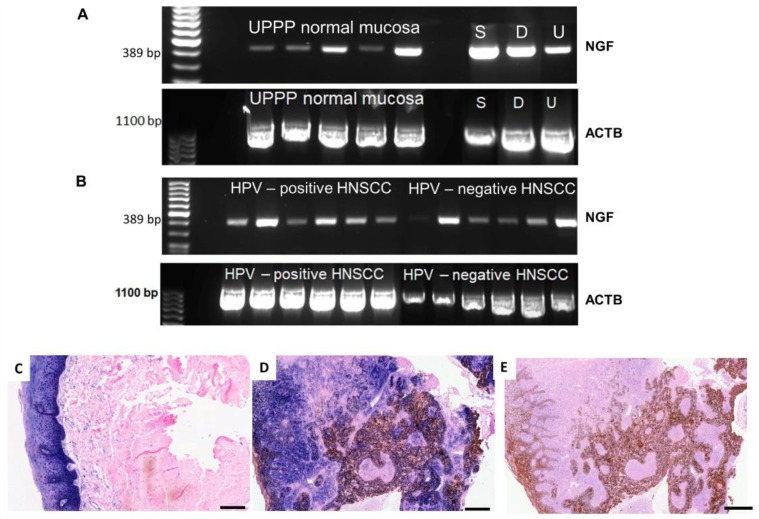
mRNA expression of nerve growth factor (NGF) and protein synthesis of NTRK1 in normal mucosa from uvulopalatopharyngoplasty (UPPP) and in HNSCC. (**A**) RT-PCR from reverse transcribed RNA representing a product of 389 base pairs of NGF and of 1100 base pairs of housekeeping gene β-actin (ACTB) in five samples of normal mucosa from UPPP and in SCC-25 (S), Detroit 562 (D) and UPCI-SCC090 (U) HNSCC cell lines. All mucosa samples contained various levels of NGF mRNA, HNSCC cell lines expressed high levels of NGF mRNA; (**B**) RT-PCR from reverse transcribed RNA representing a product of 389 base pairs of NGF and of 1100 base pairs of housekeeping gene ACTB in 6-6 samples of HPV^+^ and HPV^−^ HNSCC tumor tissue. All tumor samples contained various levels of NGF mRNA. In HPV-negative samples the housekeeping gene showed also variation; (**C**) Combined ISH of antisense riboprobe of NGF (blue) and immunohistochemistry of NTRK1 (brown) in normal mucosa from UPPP. No separate brown signal was visible because of the co-localisation of NGF and NTRK1 (dark, nearly black staining in the basal cells). In the basal cells, traces of brown color and black reaction represented both NGF and NTRK1 signals; (**D**) Combined ISH of NGF antisense riboprobe (blue) and IHC of NTRK1 (brown) in hypopharynx squamous cell carcinoma. Besides cells positive for both blue and brown signals, there were also separate brown and blue stained areas visible; (**E**) Combined ISH of control sense riboprobe (blue) and IHC of NTRK1 (brown) in hypopharynx squamous cell carcinoma. The control sense probe showed only a minimal blue background reaction. Images were taken by the TissueFaxs system, bars: 200 µm. Pink background on (**C**,**D**) is the colour of the counterstain, nuclear fast red (Red Counterstain of Ventana). The ISH was repeated on three sections of the tissue samples, a representative example is shown.

**Figure 2:**
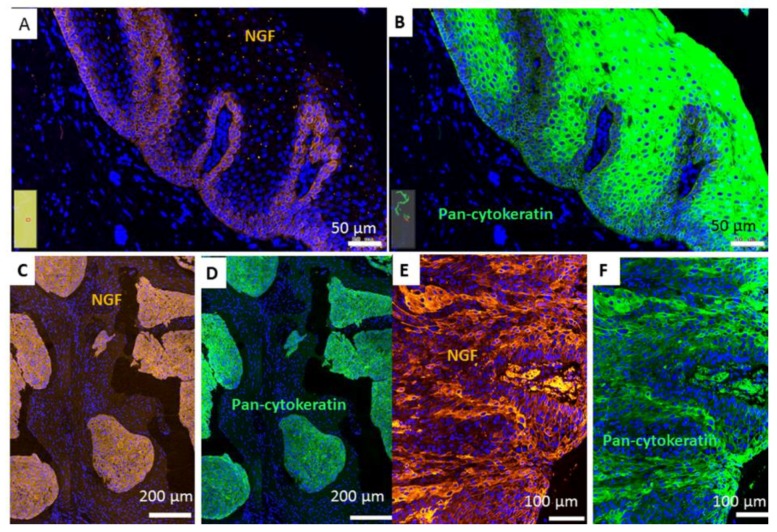
mRNA expression of NGF using a Digoxigenin-labeled antisense probe (NGF, orange or browndetected by anti-digoxigenin-rhodamin (**A**,**C**,**E**); and IHC of pan-cytokeratin (green, Alexa Fluor 488) (**B**,**D**,**F**) in normal mucosa from uvulopalatopharyngoplasty (UPPP; (**A**,**B**)), larynx (**C**,**D**) and oral (**E**,**F**) HNSCC. Images were taken by the TissueFaxs system; bars are indicated on the images. The cell nuclei in all sections were counterstained in DAPI (blue). The ISH was repeated on three sections of the tissue samples, a representative example is shown.

Using RNA isolation, reverse transcription, PCR and in situ hybridization (ISH), the mRNA expression of NGF, and using immunohistochemistry (IHC), the protein synthesis of NGF receptors, NTRK1 and p75NTR, were investigated in normal mucosa from uvulopalatopharyngoplasties (UPPP) in HNSCC tumor tissue and in HNSCC cell lines. The normal epithelium of UPPP (Figure 1A,C), HNSCC tumor cell lines (Figure 1A) and HNSCC tissue samples (Figure 1B) expressed various levels of NGF mRNA. Normal mucosa and HNSCC tissue samples included examples of high and low NGF gene expression, as well as different levels of housekeeping gene ACTB (β-actin) (Figure 1A,B). Using ISH, we attempted to identify the source of NGF-producing cells in normal oral mucosa, and in HNSCC. In the normal mucosa, the epithelial cell compartment was the main source of the NGF mRNA, while the stroma had a low and sporadic expression (Figure 1C). In addition, a gradient of NGF was observed, presenting high reactivity in basal cells and lower intensity towards suprabasal cells (Figure 1C). These results have been also confirmed by fluorescent detection of the NGF ISH signal and by simultaneous immunohistochemical detection of cytokeratin (Figure 2A,B). In HSNCC tissue, ISH evidenced that the cancer cell nests (Figure 1D and Figure 2C–F) were capable of NGF mRNA synthesis. Figure 2C–F presents a co-localization of the antisense NGF riboprobe and the pan-cytokeratin antibody in larynx and oral HNSCC tumor cell nests. 

**Figure A1:**
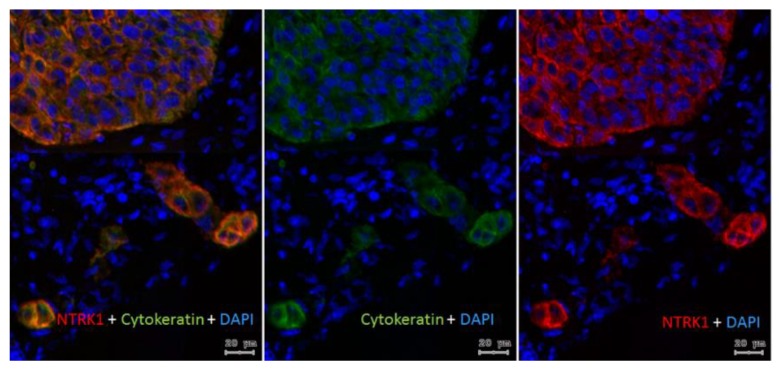
NTRK1 and Cytokeratin co-localisation; red: NTRK1, green: Cytokeratin, blue: cell nuclei counterstained, scale bars: 50 µm.

Similar to NGF, the high affinity NGF receptor, NTRK1 [27] was localized to the epithelial cell compartment of both normal epithelium and HNSCC (Figure 1C,D). In epithelium of UPPP, NTRK1 positive IHC reaction was limited to the basal cell layer (Figure 1C). In contrast, in HNSCC (Figure 1D,E) apparently all cells in the cancer cell nests were stained with NTRK1 antibody. No clear relation was found between the distribution pattern of NGF mRNA expression and NTRK1 protein synthesis. NTRK1 clearly co-localized with cytokeratin in established cancer cell nests as well as in small cytokeratin-positive groups of disseminating tumor cells (Appendix A; Figure A1). These results suggested an epithelial characteristic to both NGF mRNA expression and NTRK1 protein synthesis, which was obvious in the case of normal mucosa, and was further evidenced for HNSCC.

**Figure 3:**
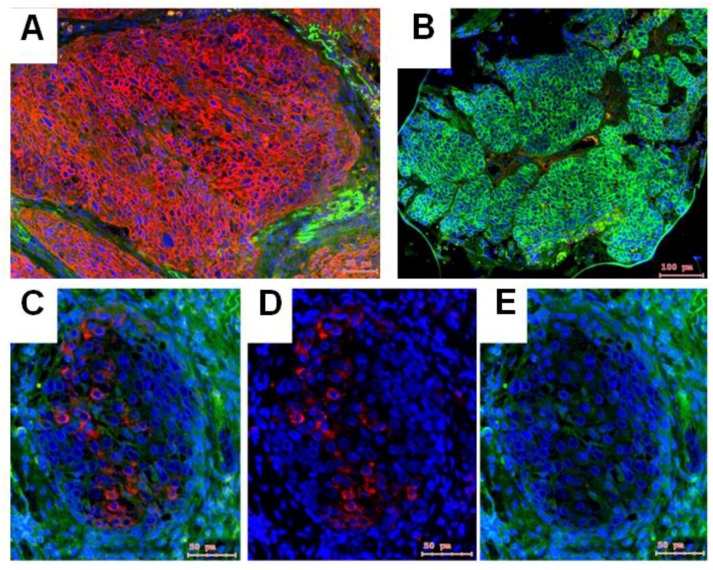

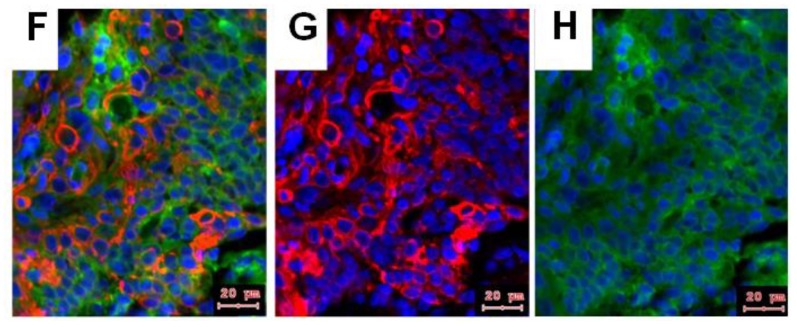
IHC staining of NTRK1 (red) and p75NTR (green) in HPV-positive (**A**,**F**–**H**) and HPV-negative (**B**,**C**–**E**) oropharynx squamous cell carcinoma (OSCC). (**A**) Combined IHC-staining of NTRK1 (red or orange on green background) and p75NTR (green) in an example of HPV-positive OSCC). In this sample NTRK1 staining was dominant in tumor cell nests and p75NTR was present only in stroma. (**B**) Combined IHC-staining of NTRK1 (red) and p75NTR (green) in an example of HPV-negative OSCC. In this sample, p75NTR-staining was detected in tumor cell nests without traces of NTRK1. (**C**–**E**) Combined (**C**) and separated IHC-staining of NTRK1 ((**D**), red) and p75NTR ((**E**), green) in an example of HPV-negative OSCC. In the tumor cell nests, NTRK1 and p75NTR were both present, but the positive cells were mutually exclusive. (**F**–**H**) Combined (**F**) and separated IHC-staining of NTRK1 ((**G**), red) and p75NTR ((**H**), green) in an example of HPV-positive OSCC. In the tumor cell nests NTRK1 and p75NTR were both present, but the NTRK1 positive cells showed weaker p75NTR staining. Images were taken by the TissueFaxs system, bars are indicated on the images. The cell nuclei in all sections were counterstained in DAPI (blue). Although the HPV^+^ or HPV^−^ nature of the cases are mentioned, the staining pattern is not necessarily related with the HPV carcinogenesis.

In contrast to NTRK1, which was broadly distributed (Figure 1D,E), about the half of the HNSCC cases did not synthesize p75NTR in their epithelial cancer cell nests (an example is visible in Figure 3A), whereas it was detected in stroma, or at the border of the cancer cell nests (Figure 3A,C). In few cases, 7 of 93 HNSCC, the opposite was found: a broad p75NTR staining in epithelial cancer cell nests (Figure 3B, green reaction), without NTRK1 staining. In cases where both NTRK1 and p75NTR were present, they were detected in mutually exclusive cells (Figure 3C–E), or the p75NTR–positive cells showed low NTRK1 reaction and vice versa (Figure 3F–H). 

### 2.2. NTRK1 and p75NTR NGF Receptors Synthesis Negative Correlate in HNSCC Tumor Specimens

UPPP and HNSCC samples were immunohistochemically investigated for the high and low affinity NGF receptors. In the available 12 UPPP samples: five (41.6%) showed positive reaction for NTRK1 and eight (66.636%) showed positive reaction for p75NTR. The staining intensity of NTRK1 was weak, while in three UPPP cases the p75NTR staining intensity was high. Five UPPP samples showed positive reaction for both of NTRK1 and p75NTR, four UPPP samples had no staining of any of these NGF receptors. NTRK1 was not present in normal mucosa without p75NTR, while in three normal mucosa samples p75NTR was not accompanied by NTRK1. 

Ninety-three HNSCC samples were available for NTRK1 and p75NTR IHC. Seventy-five of 93 (80.64%) HNSCC showed positive reaction for NTRK1 and 48 of 93 (51.61%) HNSCC showed positive reaction for p75NTR. Increased NTRK1 IHC (higher than the level of normal mucosa) was found in 73 of 93 cases (78.50%), whereas increased p75NTR IHC (at least 33% of the cells in tumor cell nests positive) was found in 20 of 93 cases (21.50%). Seven cases were negative for both of NTRK1 and p75NTR, 14 cases showed increased reaction for both of NTRK1 and p75NTR, 45 cases had only NTRK1 and no p75NTR, and 7 cases had only p75NTR and no NTRK1. 

**Figure 4:**
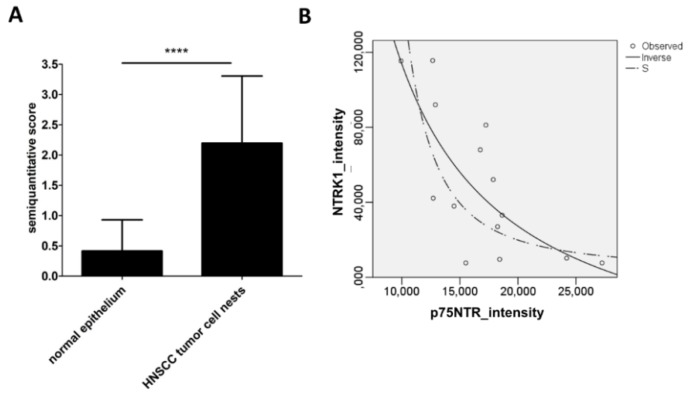
NTRK1 representation in UPPP and HNSCC specimens. (**A**) In a sample of 93 HNSCC and 12 UPPP specimens, the NTRK1 and p75NTR IHC intensity ranged no staining (0), low (score 1), middle (score 2) and high (score 3). The NTRK1 staining score was significantly (**A**) (*p* < 10^−4^ ****) higher in the cancer cell nests of HNSCC than in the normal epithelium of the UPPP samples. (**B**) In a sample of 14 HNSCC specimens containing both NTRK1 and p75NTR staining in the cancer cell nests, the staining intensity was evaluated by HistoQuest (Supplementary Information, Supplementary Methods, Figures S1–S3). The values of p75NTR intensity were plotted on the X-axis and of NTRK1 intensity on the Y-axis. The p75NTR intensities were lower. The relationship between X-Y values was modeled by SPSS^TM^ and a significant (*p* = 0.002) inverse relationship was found.

The NTRK1 IHC was significantly (Figure 4A, *p* < 10^−4^) higher in the cancer cell nests of HNSCC than in the normal epithelium of the UPPP samples. Neither NTRK1 nor p75NTR IHC showed any significant difference in any of the HNSCC localizations. In a sample of 14 HNSCC specimens containing both NTRK1 and p75NTR staining in the cancer cell nests, the staining intensity was evaluated by HistoQuest (Supplementary Information; Supplementary Methods). The values of p75NTR intensity were plotted on the *X*-axis and the ones of NTRK1 intensity on the *Y*-axis. p75NTR intensities were lower (Figure 4B). The relationship between X-Y values was modeled by SPSS^TM^ and a significant (*p* = 0.002) inverse relationship was found (Figure 4B). Correlation analysis by Spearman´s rho showed a high significant (*p* = 0.005) negative correlation between p75NTR and NTRK1 intensity (correlation coefficient: −0.7). Taken the IHC results together, in HNSCC the NTRK1 staining was high in the majority of the tumor cell nests, the tumor cells were either stained for NTRK1 or for p75NTR, in the case if both receptors were present, the cells stained with p75NTR and the ones stained with NTRK1 were mutually exclusive. 

### 2.3. Patient Survival Relation of NTRK1 and p75NTR in Human Papilloma Virus (HPV) Positive and Negative HNSCC Cases

As presented previously, both HPV-positive and -negative HNSCC tissues were capable of NGF-gene-expression (Figure 1B). HPV-positive cases were decided by IHC of the surrogate marker p16^INK4^ being in at least 66% of the tumor cells positive. Taking HPV DNA PCR analysis as the reference method, the sensitivity of p16 IHC was 78% and the specificity was 79% [28]. The p16^INK4^—based HPV evaluation was possible in 92/93 HNSCC cases. Twenty-eight cases were HPV-positive and 64 cases were HPV-negative. Overall, 84.37% of HPV-negative cases and 75% of HPV-positive cases showed increased NTRK1 staining. The staining intensity of NTRK1 in HPV-positive and -negative HNSCC did not differ significantly (*p* = 0.147 using Mann–Whitney test). In total, 53.12% of HPV-negative and 50% of HPV-positive cases were p75NTR-positive. The staining intensity in HPV-positive and -negative HNSCC did not differ significantly (*p* = 0.9 using Mann–Whitney test). The HPV carcinogenesis background did not show any relation with immunohistochemical detection of NGF receptors. 

The NTRK1 and p75NTR staining levels were not related to significant patient survival effects in Kaplan–Meier censored case survival processing where Log Rank (Mantel–Cox) pairwise comparisons were performed (Appendix B; Table A1), if all cases were processed. Because of the strong beneficial survival influence of HPV-background [25,26], HPV-positive and -negative cases were also separately processed, to eliminate the major survival influence of the HPV background. Indeed, 90% of HPV-positive patients with wild type TP53 survived two years after first contact, while 50% HPV-negative patients with altered p53 were lost within two years after first contact (own unpublished data, Log Rank (Mantel–Cox) pairwise comparison; *p* < 10^−3^). The NTRK1 level or the p75NTR presence did not show any significant patient survival influence either in HPV-positive or negative HNSCC cases. In HPV-positive cases there was a visible, but not significant tendency for lower survival rate (66.7% against 85.7%) and for shorter survival time (41 months against 62 months) if NTRK1 protein level was above the control normal tissue level. p75NTR presence or absence did not show any patient survival difference in HPV-positive cases (Appendix B; Table A1). In contrast, in HPV-negative cases, only when p75NTR high staining was accompanied by high NTRK1 staining compared with the other cases where both p75NTR and NTRK1 were low or not present, or only one of them was present, the patient survival was significantly lower (*p* = 0.013) in the Log Rank (Mantel–Cox) pairwise comparison (*n* = 64) (Appendix B; Table A1). Taken together, in HPV-positive cases an increased NTRK1 might be related with lower patient survival option, but this trend was not significant yet in the available patient collective. In HPV-negative cases the high expression of NTRK1 and the presence of p75NTR at the same time showed a statistically significant lower survival. 

### 2.4. NGF Receptors in SCC-25, Detroit 562 and UPCI-SCC090 Cells

**Figure 5:**
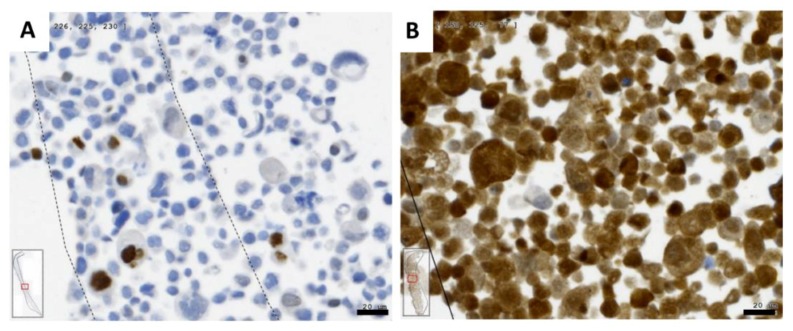
p53 (**A**); and p16^INK4^ (**B**) representation in UPCI-SCC090 cells. Cells (2–4 × 10^6^) were collected by centrifugation and embedded as cell pellet in agarose followed by embedding in paraffin (Supplementary Information, Supplementary Methods). Five micrometer thick sections have been cut from the cell pellets, stained using p53 and p16^INK4^ specific antibodies, and photographed using the TissueFaxs system. Bars: 20 µm.

**Figure A2:**
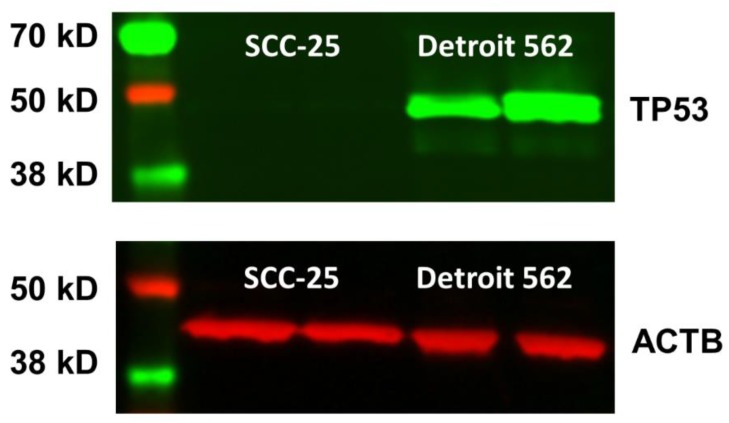
Western blot of p53 protein (green) in SCC-25 and Detroit 562 cells, beta-actin (red) (ACTB) was used as loading control. Protein signal were detected by near infrared fluorescence.

We described before that NGF gene expression is confined to normal and malignant epithelial cells and both normal and malignant epithelial cells might express NGF receptors. In HNSCC the expression pattern was not different in HPV-positive and -negative tissue samples, both NGF receptors could have been present in tumor cell nests. In a further step, we used SCC-25, Detroit 562 and UPCI-SCC090 HNSCC cell lines for investigation of NGF receptors; the NGF gene expression has been confirmed before (Figure 1A). SCC-25 cells were originally isolated from the primary tumor of a patient with tongue carcinoma [13,29]. SCC-25 cells are from primary oral SCC, maintained in in vitro cultures, might be also xenografted, but they grow only in severe combined immunodeficiency (SCID) mice and not in athymic mice. SCC-25 cells did not show metastatic potential in mouse xenograft models [30]. In vitro, SCC-25 cells were published to be radioresistant [31]. SCC-25 cells contain a deletion and a frame shift in codon 209 of the protein coding region of the *TP53* gene and synthesize a truncated p53 protein, which probably quickly degrades (own Sanger sequencing results, and Western blot with mouse monoclonal DO-7 p53 N-terminal specific antibody; Appendix C, Figure A2). Detroit 562 cells demonstrated radio and chemoresistance [32,33], and it is a potential metastatic cell line in xenograft models [34]. Detroit 562 cells were originally isolated from the malignant pleural effusion of an OSCC [35,36]. A frequent gain of function mutation R175H of *TP53* gene is contained in Detroit 562 cells [37], which has been confirmed by us utilizing Sanger sequencing. Both SCC-25 and Detroit 562 cells were HPV-negative [37]. The UPCI-SCC090 cell line has been established by Robert Ferris and co-workers [38]. UPCI-SCC090 cells contain genome integrated HPV-16 DNA. The UPCI-SCC090 cells synthesize both p16^INK4^ surrogate HPV marker protein and E6, E7 HPV oncogene product proteins [38]. Ferris et al. [38] published a wild-type tumor suppressor *TP53* gene in this cell line. In our hands, the protein coding region of UPCI-SCC090 *TP53* gene contained the following miss-match nucleotide alterations in the following codons: D49V, P72R, T150A, and R209G. None of these have been reported to have any consequences in the p53 protein function, and they might be polymorphisms similar to P72R. We have done IHC analysis in agarose and paraffin embedded UPCI-SCC090 cells using diagnostic antibodies against p53 protein and p16^INK4^. p53 protein showed a typical scattered distribution (Figure 5A), which is usually observed in patients with no p53 mutations [39], and p16^INK4^ was homogeneously distributed in all cells (Figure 5B). 

**Figure 6:**
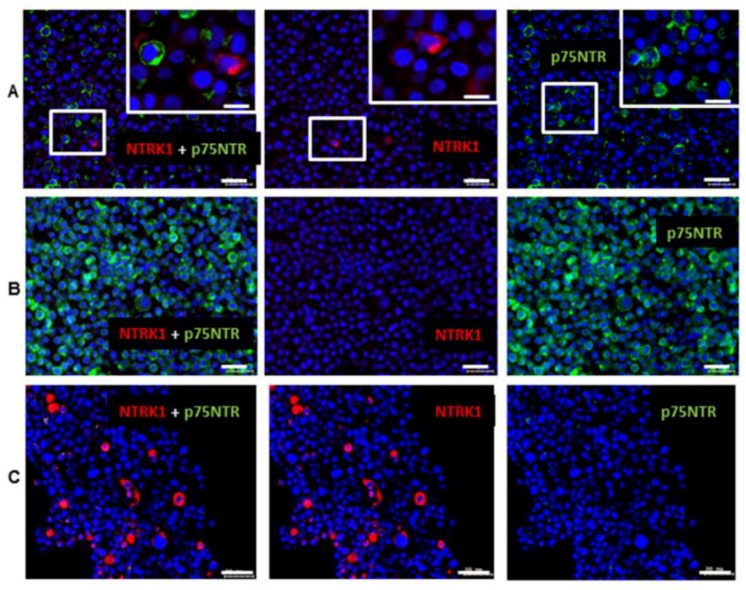
NTRK1 (red) and p75NTR (green) representation in: SCC-25 cells (**A**); Detroit 562 cells (**B**); and UPCI-SCC090 cells (**C**). Cells (2–4 × 10^6^) were collected by centrifugation and embedded as cell pellet in agarose followed by embedding in paraffin (Supplementary Information, Supplementary Methods). Five micrometer thick sections have been cut from the cell pellets, stained using NTRK1 and p75NTR specific antibodies, and photographed using the TissueFaxs system. The NGF receptors are shown together and separated. Scale bars: 50 µm in the images and 10 µm in the insets, blue: cell nuclei counterstained with DAPI.

The IHC staining of low and high affinity NGF receptors was also investigated in formalin fixed and paraffin embedded pellets of the above-introduced cell lines. SCC-25 cells were heterogeneous containing the majority of the cells without any NGF receptors, but also scattered cells expressing either low NTRK1 or well visible p75NTR. Both receptors were not present together (Figure 6A, in the figure insets the separate expression of NGF receptors is well visible). All Detroit 562 cells were p75NTR positive and they did not contain any NTRK1 (Figure 6B). The UPCI-SCC090 cells culture contained cells without any NGF receptors, and also frequently cells with high expression of NTRK1 were present, while low p75NTR positive cells were very rare (Figure 6C).

### 2.5. Cell Survival Effects of NGF Treatments in SCC-25, Detroit 562 and UPCI-SCC090 Cells after Cell Cycle Arrest

The next task was to test the hypothesis that NGF and any of its receptors might contribute to cell survival after cell cycle arrest. Cell cycle arrest after DNA damage is a common challenge for tumor cells in several therapeutic settings. Mitomycin C (MMC) is widely used in DNA-crosslinking/damage and cell cycle arrest studies [40], it preferentially eliminates proliferating cells, while showing less interference with the viability of G1-arrested cell populations [41] and it also does not affect cell migration [42]. A 30 min 10 µg/mL MMC-treatment in SCC-25 HNSCC cells enabled an up to 96 h cell cycle arrest, and there was no cell division within this time in this cell line [43]. In addition to cell cycle arrest, the MMC response was reported to induce cell death as both apoptosis and necrosis [44].

**Figure 7:**
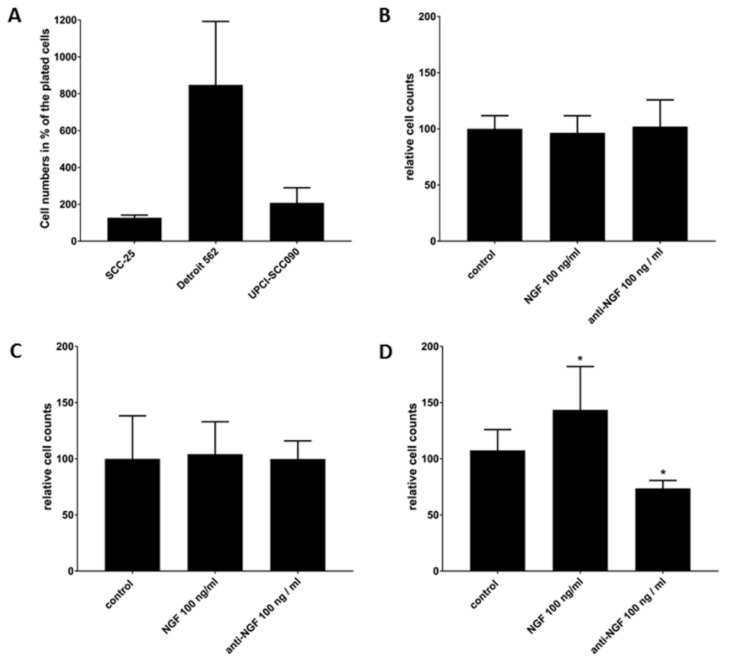
(**A**) Effects of MMC 96 h after a single 10 µg/mL treatment for 30 min in SCC-25, Detroit 562 and UPCI-SCC090 cells, the plated 6.7 × 10^4^ cells were considered as 100%. SCC-25 cell culture remained at the plated level, Detroit 562 cell culture expanded and UPCI-SCC090 cell culture doubled after 96 h. Relative cell counts in SCC-25 (**B**), Detroit 562 (**C**) and UPCI-SCC090 (**D**) cells after two times 48 h of NGF and neutralizing anti-NGF antibody treatments after a single MMC-induced cell cycle arrest. Control (MMC-treated but not NGF or anti-NGF treated) cell counts were taken as 100% by all cell lines. Cell counts were analyzed by Graphpad Prism 7.01, were normal distributed and the mean values and SD of NGF and anti-NGF treated cells were compared with the control using one-way analysis of variance (ANOVA) and Dunnett’s multiple comparisons test. * *p* < 0.05.

The used HNSCC cell lines were plated at 6.7 × 10^4^ cells/mL in serum-free culture media, the whole protein content of serum was replaced by serum albumin [32,43]. The cells were allowed to grow for 72 h, and then were treated with 10 µg/mL MMC for 30 min followed by treatments for two times 48 h with 100 ng/mL NGF [45], or 100 ng/mL anti-NGF neutralizing antibody [46]. At the end of the whole experiment the SCC-25 culture contained 126.86 ± 14.92% of the originally plated cells, the Detroit 562 culture contained 848.26 ± 344.43% and the UPCI-SCC090 culture contained 207.71 ± 82.48% (Figure 7A). The SCC-25 cell culture was not only cell cycle arrested by a single short MMC treatment, but also contained 20.56 ± 7.92% of cells stained positive with trypan blue, indicating incapacitated cell membrane functions, characteristic for necrosis or late apoptosis. The fraction of dead cells did not differ significantly after treatments with NGF or anti-NGF (*p* = 0.74–0.99 by Kruskal–Wallis test). The average number from three biological repeats of control cells (96 h after single 10 µg/mL 30 min MMC treatment) was defined as 100%, and the cell numbers from NGF and anti-NGF treated cells (from three biological repeats) were compared to the control by Kruskal–Wallis test. There was no significant difference between the control and the treated cells in case of SCC-25 using any of the treatments (*p* > 0.99, Figure 7B). The Detroit 562 cell culture was still expanding after 96 h of the single short MMC treatment, and contained 8.07 ± 3.63% of cells stained positive with trypan blue, which was comparable with routine cell culture conditions. The fraction of dead cells did not differ significantly after treatments with NGF or anti-NGF (*p* = 0.1–0.74 by Kruskal–Wallis test). The average number from three biological repeats of control cells (96 h after single 10 µg/mL 30 min MMC treatment) was defined as 100%, and the cell numbers from NGF and anti-NGF treated experiments (from three biological repeats) were compared to the control by Kruskal–Wallis test. There was no significant difference between the control and the treated cells by Detroit 562 cells using any of the treatments (*p* > 0.99, Figure 7C). The UPCI-SCC090 cell culture doubled after the single short MMC treatment, compared to the plated cell numbers (Figure 7A), and contained 6.09 ± 7.04% of cells stained positive with trypan blue, which was comparable with routine cell culture conditions. The fraction of dead cells did not differ significantly after treatments with NGF, or anti-NGF (*p* = 0.16–0.48 by Kruskal–Wallis test). The average number from three biological repeats of control cells (96 h after single 10 µg/mL 30 min MMC treatment) was defined as 100%, and the cell numbers from NGF and anti-NGF treated experiments (from three biological repeats) were compared to the control by Kruskal–Wallis test. NGF (100 ng/mL) provided a significant increase (*p* = 0.008, Figure 7D) and 100 ng/mL anti-NGF neutralizing antibody assured a significant decrease (*p* = 0.018, Figure 7D) of the cell numbers. These results are summarized in Table 1.

## 3. Discussion

Based on available references [47,48] NGF was hypothesized as a potential trophic factor available from epithelial sources in HNSCC tissue. In HNSCC tumor specimens and in UPPP controls, NGF mRNA was detected by RT-PCR and ISH in epithelial cells including basal cells of normal squamous cell epithelium and malignant tumor cells in cancer cell nests. Over 80% of HNSCC specimens showed positive reaction for high affinity NGF receptor, NTRK1, and nearly all of them a high positive reaction in cancer cell nests. In contrast to NTRK1, the low affinity receptor p75NTR was present in 51.61% of HNSCC samples. p75NTR protein synthesis was not more frequent in cancer cell nests than in normal mucosa, but NTRK1 staining was significantly higher in the cancer cell nests of HNSCC than in the normal epithelium. Interestingly, high significant (*p* = 0.005) negative correlation was found between p75NTR and NTRK1 staining intensity (correlation coefficient: −0.7). Even if both NTRK1 and p75NTR were present in the tumor cell nests, they were mutually exclusive, meaning that NTRK1^+^ and p75NTR^+^ cells are two separate cell populations of the HNSCC tumor cell nests; moreover, this finding could be extended to cultured cell lines, as revealed on Figure 6.

Similar to previous findings in prostate cancer [49,50,51], cancer cell nests containing both NGF and NTRK1 suggested an important autocrine tumor maintenance function of this system in HNSCC. We refer to a previously published issue that the NTRK1-NGF axis can be a survival system, which rescues cells from cell death [17].

In HNSCC, the HPV-carcinogenesis has been published to contribute to preferred patient survival [25,26] compared to HPV-negative background, which has been confirmed by our data as well. The HPV carcinogenesis background did not show any relation with *NGF* gene expression or with IHC detection of NGF receptors. Patient survival data were related to NTRK1 and p75NTR staining levels, and none of the NTRK1 and p75NTR staining levels did show any significant survival effects in Kaplan–Meier censored case survival processing. Additionally, HPV-positive and -negative cases were also separately processed. p75NTR presence or absence did not show any survival difference in HPV-positive cases, as well as the NTRK1 levels were also not related with a significant survival influence. A significant effect has been seen in HPV-negative cases, when p75NTR high staining was accompanied by high NTRK1 staining in the tumor cell nests, which was related with significantly lower patient survival compared with cases where both p75NTR and NTRK1 were low or not present, or only one of them was present. These data suggest that the positive HPV detection is related with patient survival benefit, as confirmed by the current data and by published references [25,26], and it is at the moment the strongest considerable patient survival influencing factor in HNSCC. In HPV^+^ cases the neurotrophin receptors were not related with significant survival effects. These data indicate that the effects of neurotrophin receptors levels might be secondary to the strong beneficial effects related with still unclear biological mechanisms associated with high p16^INK4^ IHC. 

The current statistical data of a limited patient collective were compared with experimental results on three HNSCC cell lines. All three cell lines produced sufficient amounts of NGF. The neurotrophin receptors expression was scattered in SCC-25 cells, and the majority of the cells were without any NGF receptors. In contrast, all Detroit 562 cells were p75NTR positive, but they did not contain any NTRK1. The UPCI-SCC090 cells culture frequently contained cells with high expression of NTRK1, while p75NTR positive cells were very rare. In these three cell lines a single 30 min 10 µg/mL MMC-treatment in serum and growth-factor-free conditions, which enabled an up to 96 h cell cycle arrest in SCC-25 cells, was used as an anti-proliferative challenge. The MMC-challenge was followed by two-times 48 h NGF or neutralizing NGF antibody treatment. Both the NTRK1-positive UPCI-SCC090 culture (at 2-fold) and the p75NTR-positive Detroit 562 culture (at 8-fold) increased after the MMC-challenge without NGF treatment (based only on the self-production of NGF), and the SCC-25 culture remained arrested. Interestingly, SCC-25 and Detroit 562 cells did not react on given 100 ng/mL NGF or 100 ng/mL neutralizing anti-NGF antibody, while UPCI-SCC090 cells did. 

The received results allow the following conclusions. NGF might improve cell survival after a cell cycle arrest in tumor cell nests (or cell culture), which contain sufficiently high number of cells with NTRK1. High expression of p75NTR most optimally in all cells of the culture (as by Detroit 562) does not lead to cell survival increase after NGF treatment or to cell survival reduction after NGF neutralization. Nevertheless, p75NTR is still involved in cell survival support in Detroit 562 cells, without any relation to NGF. This, most probably NGF-independent mechanism was the one, which allowed an eight-fold cell culture expansion after a single MMC challenge. This effect of p75NTR might probably work through p21(WAF1), which it also contributes to drug resistance, as published by Verbeke et al. [23].

The clinical and experimental data of this study, taking also published references into consideration, delivers the following outcomes:

- HPV positivity is a primary factor, which is related with HNSCC patient survival benefit. NGF is expressed both in HPV^+^ and HPV^−^ HNSCC, neurotrophin receptors do not show a HPV-specific distribution.
- In HPV^−^ HNSCC, high levels of both NTRK1 and p75NTR might be related with significant lower patient survival. NGF seems to be sufficiently produced by normal epithelial cells and by tumor cell nests. Most importantly the co-expression of NTRK1 and p75NTR can be observed at tumor cell nest or at whole culture level, but in fact, it means separated NTRK1- and p75NTR-positive cells.
- It seems that two independent effects are needed to achieve a patient survival circumvention influence of the neurotrophin receptors in HNSCC: the first, is the NGF dependent tumor cell survival contribution of NTRK1, which is exemplified in UPCI-SCC090 cells, the second is the NGF-independent, probably p21(WAF1)-based [23] therapy resistance contribution of p75NTR, which is exemplified in Detroit 562 cells. As mentioned above, these two independent effects are also physically separated in NTRK1 and p75NTR positive cell populations.

The NGF expression and neurotrophin receptor synthesis do not explain the patient survival benefit of HPV^+^ HNSCC patients, and do not contribute to the mechanistic understanding of the phenomenon of the good prognosis of HPV-positivity in HNSCC. The NGF-neurotrophin receptor axis might be a negative risk contributor to patent survival, probably weaker than the positive effects of HPV-positivity. 

As reviewed by Tomellini et al., p75NTR can cooperate with other receptors and modify signaling pathways, by which it is able to interact both in synergistic and antagonistic ways. p75NTR, does not have an independent intrinsic enzymatic activity, it acts more as modifier of other signaling pathways [21], and as reported, it might be responsible for drug resistance [23]. At the same time, the NGF and NTRK1 receptor form a previously unknown important autocrine epithelial survival axis in HNSCC, which might be active in cell survival mechanisms following chemotherapeutic treatments of DNA crosslinking agents as cisplatin or MMC. The suggestion of NGF and NTRK1 autocrine loop in HNSCC is absolutely novel. Nevertheless, similar findings from other oncological fields support the relevance of this issue, as for example in breast cancer the formation of NGF and NTRK1 autocrine axis was evidenced as well [52]. In prostate cancer [53], the development of NGF/NTRK1 or BDNF/NTRK2 autocrine signaling pathways is an escape mechanism, from both the androgen control and the paracrine dependence of stromal cells produced neurotrophins. The strength of our study is that we clearly evidence the NGF and NTRK1 co-localization with combined IHC and ISH methods. Similar to HNSCC tissue samples, the p75NTR and NTRK1 receptors were in different cells even in cultured cell lines, also if both receptors were expressed. 

## 4. Materials and Methods

### 4.1. Patient Samples, Immunohistochemistry

The procedures followed were in accordance with the ethical standards of the committee on human experimentation of the institution and in accord with the Helsinki Declaration of 1975 as revised in 1983. Permission was obtained from the local ethics committee to collect pretreatment biopsy samples for molecular biological investigation, paraffin embedding, sectioning and immunohistochemical analysis (Reference Number: UN4428 303/4.14). Informed consent was obtained from all patients. Neurotrophin receptor determinations were performed in 93 randomly selected specimens of incident locally advanced HNSCC treated between March 2010 and October 2017 at the Department of Otorhinolaryngology—Head & Neck Surgery, Medical University of Innsbruck. Clinical data are summarized in Appendix B, Table A2. As a control, 12 normal mucosa tissue samples derived from uvulopalatopharyngoplasties (UPPP) in patients with sleep apnea syndrome were included. Pretreatment tumor samples were obtained during diagnostic panendoscopy. Patient samples were paraffin embedded, sectioned and immunostained as described in Supplementary Information, Supplementary Methods, antibody information is provided in Supplementary Information, Supplementary Methods Table S1.

### 4.2. In Situ Hybridization

In situ hybridization was performed on 5 µm paraffin sections in a Ventana Discovery Classic immunostainer (Tucson, AZ, USA) using Ribomap kit and Bluemap kit (Ventana) utilizing digoxigenin (DIG) labeled riboprobes described in Supplementary Information, Supplementary Methods. For antigen retrieval CC1 buffer (mild) and Protease 3 (16 min) were used. DIG-labeled riboprobe (200 ng/mL) and unlabeled sheared salmon sperm DNA (160 µg/mL) (Ambion, Fisher Scientific, Vienna, Austria) were added in Ribohybe (Ventana) (100 µL) on each slide. The salmon sperm DNA was used to compete the cell nuclear DNA binding of the DIG-labeled riboprobe. The probe hybridization was performed at 66 °C for three hours followed by stringency 2xSCC washes, 3 × 8 min at 70 °C, as suggested by DIG “In situ Application Note No. 1” (Roche Diagnostics, Mannheim, Germany, 2012) [54]. The hybridization signal was detected by anti-DIG Fab fragments (alkaline phosphatase or rhodamin coupled) purchased from Roche Life Sciences, and by the Bluemap kit (Roche, Ventana) as instructed by the providers. If the alkaline phosphatase was used, the BCIP, NBT substrate time was 3 h. The cell nuclei were counterstained in red by Red Counterstain II (Ventana) or by DAPI in case of the fluorescence system. If using fluorescence, the in situ hybridization was directly followed by the incubation with primary and secondary antibodies as described in the immunohistochemistry section in Supplementary Information, Supplementary Methods. The antisense riboprobe showed a specific reaction, while the control probe did not react specifically.

### 4.3. Image Analysis of Immunohistochemistry and In Situ Hybridization

The immunostained and riboprobes reacted sections were digitalized at 20× magnification utilizing a TissueFaxs Plus System coupled onto a Zeiss^®^ Axio Imager Z2 Microscope (Jena, Germany). Regions of interest were then acquired using the TissueFaxs (TissueGnostics^®^, Vienna, Austria). The intensity of signals localized onto the affixed sections was then evaluated using HistoQuest^®^ (TissueGnostics) software. Details of the intensity quantification are given in Supplementary Information, Supplementary Methods.

Alternatively, p75NTR and NTRK1 immunostaning were scored (0): no staining; low (1): under 30% of cells positive; middle (2): 30–66% of cells positive; and high (3): more than 66% of cells positive in cancer cell nests) and Mann–Whitney test was used to detect differences between HNSCC and UPPP. p75NTR staining was also evaluated as staining present (1) or absent (0). 

### 4.4. Cell Lines

SCC-25 and UPCI-SCC090 cells were acquired from the German Collection of Microorganisms and Cell Cultures (DSMZ, Braunschweig, Germany, DSMZ no.: ACC 617). SCC-25 cells were cultured in DMEM/F12 medium. UPCI-SCC090 [38] cells were cultured in EMEM medium supplemented with 10% FBS, 2 mM l-glutamine, 100 units/mL penicillin and 100 μg/mL streptomycin [14,15,32]. Detroit 562 cells were purchased from Cell Lines Service (CLS, Eppelheim, Germany) and were cultured in EMEM medium supplemented with 10% FBS, 2 mM l-glutamine, 100 units/mL penicillin and 100 μg/mL streptomycin. For experimental purposes, cells were cultured in an albumin-containing medium where serum proteins were replaced by 4.4 g/L bovine serum Page: 13 Detected space and/or hyphen in reference call-out. This is not recognized, space should be removed and hyphen changed to en dash. Albumin from PAA Laboratories (Pasching, Austria). 

### 4.5. Evaluation of the NGF Receptors in Cultured Cell Lines 

Routinely cultured cell lines (2–4 × 10^6^) were collected by centrifugation and embedded as cell pellet in agarose as published before [55], modified as follows: Cells were harvested by centrifugation at 290× *g* for 10 min at 4 °C, and the resulting pellet was fixed in 10 mL neutral buffered 4% formaldehyde solution (Flintsbach am Inn, Germany). After fixation, the cells were centrifuged by 400× *g* for 10 min at room temperature. The cell pellet was resuspended in 300 µL PBS, transferred to Eppendorf tube (1.5 mL), and kept on ice. Low melting point agarose (with gelling temperature point 34–37 °C) was prepared in PBS as 3% solution in labor glassware by microwave warming and it was equilibrated in a thermoblock to 65 °C for at least 30 min. The 300 µL PBS—cell suspension was also equilibrated to 65 °C for not more than 10 min. Six hundred microliters melted equilibrated agarose was pipetted to the cell suspension, followed by spinning at 2000× g for 5 min at room temperature. After that, the tube was placed on ice, the cell pellet was trimmed and it was placed in embedding cassette. The cell pellet in the cassette was stored in PBS containing 0.05–0.1% sodium azide until embedded in paraffin (Supplementary Information, Supplementary Methods). 

Similar to the tissue sections, from the cell pellets, 5 µm thick sections have also been cut. The cell sections did not contain any overlaps, the cells were distributed. The cell sections were stained immunohistochemically exactly ident with the tissue sections. The % of NTRK1 and p75NTR positive cells was identified after scanning the sections in the TissueFaxs system and evaluating with Histoquest software (Supplementary Information, Supplementary Methods). 

### 4.6. Cell Treatments 

For the treatment with 100 ng/mL NGF or 100 ng/mL anti–NGF neutralizing antibody (RnD Systems, Minneapolis, MN, USA), 1.5 mL/well cell suspension of 6.7 × 10^4^ cells/mL were plated in 12-well plates (Falcon^TM^, Durham, NC, USA) in serum-free, albumin containing medium [43] and cultured for 72 h. After that, the cells were washed with PBS and incubated with 10 µg/mL Mitomycin C (Sigma-Aldrich^®^, St. Louis, MO, USA) in serum-free albumin-containing medium for 30 min at 37 °C ensuring cell cycle arrest [42]. Then the cells were washed twice with albumin-containing medium and subsequently treated with albumin-containing medium for two times 48 h supplied with 100 ng/mL recombinant human NGF [45] or with 100 ng/mL anti NGF neutralizing antibody (RnD Systems) for altogether 96 h. After completion of treatments, the cells were used for cell counting with trypan blue staining (Sigma, Darmstadt, Germany) in a Neubauer chamber (Paul Marienfeld GmbH & Co. KG, Lauda-Königshofen, Germany).

### 4.7. RNA Isolation and PCR

For RNA isolation, 2–4 × 10^6^ cells or 2–3 mm tissue slices were collected and lysed in 1 mL TRIzol^®^ Reagent (Ambion^®^, Life technologies™, Carlsbad, CA, USA), and RNA was isolated as instructed by the manufacturer of TRIzol. RNA concentrations were determined by photometric measurements (BioPhotometer plus 6132, Eppendorf, Germany). Total RNA was reverse transcribed by M-MuLV Reverse Transcriptase (GeneON, Ludwigshafen am Rhein, Germany) in a MyiQ™ cycler (BIO-RAD Laboratories, Inc., Hercules, CA, USA) following the manufacturer’s instructions. PCR of cDNA transcripts was performed in a MyiQ™ cycler (BIO-RAD Laboratories, Inc., USA) using Go-Taq master mix (Promega, Madison, WI, USA) and the following forward: 5′-CAC ACT GAG GTG CAT AGC GT-3′ and reverse: 5′-TGA TGA CCG CTT GCT CCT GT-3′ primers for NGF, and forward: ACCCTGAAGTACCCCATCGA; reverse: TGTCACCTTCACCGTTCCAG for the housekeeping gene ACTB. The primers were synthesized by Invitrogen™ (Darmstadt, Germany). The PCR setup and the cycling conditions were instructed in the manual of Go-Taq. PCR products were electrophoresed in 1% agarose run in Tris-Acetate-EDTA buffer for one hour at 100 volts. Gels were photographed in an Azure C500 (Azure Biosystems, Dublin, CA, USA). 

## 5. Conclusions

NGF is produced in sufficient amounts in normal oral epithelium and in tumor cells of HNSCC, including cultured cell lines. The basal cells of normal oral mucosa synthesize both NTRK1 high affinity and p75NTR low affinity receptors. The synthesis of these two receptors is separated to two cell populations in the HNSCC tumor cell nests: high levels of NTRK1 and p75NTR were not present in the same cells of HNSCC tumor tissue and cultured cell lines. NGF was able to transduce a survival signal via NTRK1, if sufficient number of high NTRK1^+^ cells were present in the culture, but not via p75NTR, which also confirmed that p75NTR owes its signaling ability to its association with other cytoplasmic partners [21]. It seems that, in the heterogenic HNSCC tumor cell nests and cultured cell lines, several survival strategies are present at the same time: one of them is the autocrine NGF–NTRK1 system, and a possible other is the NGF-independent (since p75NTR^+^ cells have low or no NTRK1) system, which offer a therapy resistance over p21/WAF1. More possible mechanisms at the same time ensure multiple therapy resistance options for HNSCC tumor cell nests.

## Figures and Tables

**Table 1:** Effects of NGF and anti-NGF neutralizing antibody treatments on the cell numbers in SCC-25, Detroit 562 and UPCI-SCC090 cells after cell cycle arrest and treatments. Cell counts displayed as cells 1 × 10^4^/mL. From all cell lines 6.7 × 10^4^ cells/mL were plated in serum-free culture media.

Cell Lines	Untreated		NGF 100 ng/mL		Anti-NGF 100 ng/mL		Repeats
	Trypan-Blue Negative	Trypan-Blue Positive	Trypan-Blue Negative	Trypan-Blue Positive	Trypan-Blue Negative	Trypan-Blue Positive	
SCC-25	8.5 ± 1	1.7 ± 0.7	8.2 ± 0.6	1.1 ± 0.1	8.7 ± 0.8	1.2 ± 0.5	6
Detroit 562	56.8 ± 23.1	4.6 ± 2.1	59.0 ± 6.7	2.0 ±1.1	56.7 ± 3.75	2.4 ±1.4	6
UPCI-SCC090	13.9 ± 5.5	0.8 ± 1.0	19.9 ± 1.5	0.6 ± 0.2	10.2 ± 0.3	0.1 ± 0.1	6

## Supplementary Materials

The following are available online at http://www.mdpi.com/1422-0067/19/6/1771/s1.

## Abbreviations

ACTB	Beta-actin gene
BCIP	bromo-chlor-indol-phosphate
BDNF	brain-derived neurotrophic factor
DAPI	4′,6-Diamidin-2-phenylindol
DIG	digoxigenin
EDTA	Ethylene diamine tetra acetate
EMT	epithelial-to-mesenchymal transition
HNSCC	head and neck squamous cell carcinoma
HPV	human papilloma virus
LCR	long control region
LNGFR	Low-affinity Nerve Growth Factor
MMC	mitomycin C
NBT	Nitro-blue-tetrazolium
NGF	nerve growth factor
NTRK	neurotrophic receptor tyrosine kinase
OSCC	oral squamous cell carcinoma
P75NTR	p75 neurotrophin receptor
PBS	Phosphate buffered saline
PCR	Polymerase chain reaction
SCID	severe combined immunodeficiency
Trk	tropomyosin-related kinase
UPPP	uvulopalatopharyngoplasty

## Appendix A

A double staining was performed using anti-NTRK1 rabbit monoclonal antibody (detected in red) and mouse monoclonal pan-cytokeratin (detected in green) in larynx SCC. The cytokeratin and NTRK1 staining reactions were comparable, suggesting that the NGF receptor (NTRK1) is synthesized in epithelial (cytokeratin-positive) tumor cells. The immunohistochemical reactions are shown together and separately.

## Appendix B

Comparison of patient survival effects of NTRK1 and p75NTR immunohistochemical staining levels in HPV-negative and -positive HNSCC. Survival rate is the per cent of patients alive up to 96 months out of all patients. Survival time is the estimated mean ± std. error. The survival processing has been censored. The majority of the cases were not observed for 96 months.

In HPV-negative cases, the presence of p75 showed lower survival than its absence, but the difference in the Log Rank (Mantel–Cox) pairwise comparison was not significant. In the HPV-positive cases, there was no significant difference in survival regarding NTRK1 or p75NTR staining or their combination. Interestingly, in HPV-negative cases (*n* = 64) where p75NTR high staining was accompanied by high NTRK1 staining compared with the other cases where both stainings were low or not present, or only one of them was present, the patient survival was significantly lower (*p* = 0.013) in the Log Rank (Mantel–Cox) pairwise. Although the number of cases in the group where p75NTR high staining was accompanied by high NTRK1 staining was low (*n* = 10), the other group contained 54 cases, the observation time between years 2010 and 2017 was short, as well as for many cases the individual observation time was also too short and the survival processing must have been censored, there was a clear significant effect. Nevertheless, a random influence can never be ruled out by low numbers of cases. Taken together, p75NTR, especially if combined with high NTRK1 staining, was related with poor survival in HPV-negative cases.

**Table A1:** Comparison of patient survival effects of NTRK1 and p75NTR immunohistochemical staining levels in HPV-negative and -positive HNSCC.

Analyzed Cases	Investigated Question	Compared Conditions	Cases	Survival Rate, % * in up to 96 Months	Survival Time, Months (Mean ± Std. Error)	Survival Effect	*p*-Value by Log Rank (Mantel–Cox) Pairwise over Strata Comparison
ALL	NTRK1 level	NTRK1 level at control	18	66.6%	47.6 ± 8.77	No significant effect	*p* = 0.703
		NTRK1 level above control	75	60.0%	52.86 ± 5.93		
HPV-negative	NTRK1 level	NTRK1 level at control	10	60.0%	43.6 ± 11.26	No significant effect	*p* = 0.997
		NTRK1 level above control	54	57.4%	51.58 ± 6.83		
HPV-positive	NTRK1 level	NTRK1 level at control	7	85.7%	62.0 ± 11.63	No significant effect	*p* = 0.388
		NTRK1 level above control	21	66.7%	41.03 ± 5.68		
ALL	p75NTR reaction	negative	45	64.4%	47.87 ± 5.14	No significant effect	*p* = 0.413
		positive	48	58.3%	50.39 ± 7.39		
HPV-negative	p75NTR reaction	negative	30	63.3%	47.86 ± 6.20	No significant effect	*p* = 0.279
		positive	34	52.9%	45.75 ± 8.50		
HPV-positive	p75NTR reaction	negative	14	71.4%	45.22 ± 7.64	No significant effect	*p* = 0.995
		positive	14	71.4%	50.6 ± 9.30		
ALL	combined high NTRK1/p75NTR	NTRK1/p75NTR are NOT combined	79	63.3%	56.84 ± 5.77	No significant effect	*p* = 0.139
		Sign. NTRK1/p75NTR combined	14	50.0%	29.25 ± 7.18		
HPV-negative	combined high NTRK1/p75NTR	NTRK1/p75NTR are NOT combined	54	61.1%	56.41 ± 6.74	Significant worse survival if both NTRK1 and p75NTR are high	*p* = 0.013
		Sign. NTRK1/p75NTR combined	10	40.0%	17.36 ± 7.37		
HPV-positive	combined high NTRK1/p75NTR	NTRK1/p75NTR are NOT combined	24	70.8%	48.17 ± 7.50	No significant effect	*p* = 0.552
		Sign. NTRK1/p75NTR combined	4	75.0%	44.50 ± 6.72		

Sign.: significant, level above the control.

**Table A2:** Patient Data.

**Tissue Type**	**Number of Cases**	**%**
HNSCC	93	88.6
UPPP epithelium	12	11.4
**Tumor_Localization**	**Number of Cases**	**%**
oral	14	15.0
nasopharynx	4	4.3
oropharynx	39	41.9
larynx	21	22.6
hypopharynx	13	14.0
other	2	2.15
Total	93	100.0
**Gender**	**Frequency**	**Percent**
Male	85	90.9
Female	20	19.1
Total	105	100.0
**HPV**	**Frequency**	**Percent**
Positive	28	30.4
Negative	64	69.6
Total	92	100.0

Mean age of patients: 60.17 ± 13.32 years; minimum: 28; maximum: 91.

## Appendix C

### Appendix C.1. TP53 mRNA Sequencing and Protein Results in SCC-25 Cells

*TP53* mRNA amplification and sequencing in SCC-25 cells sequenced until 900 bases, which was ensured as reliable by the service provider. Explanations: Query: sequence from SCC-25 cells; Subject: database reference sequence: NM_001126112; Red label: changed sequence in SCC-25 cells; green label: original database sequence. Y is “C” or “T”.

Query 1  CATTTTCAGACCTATGGAAACTACTTCCTGAAAACAACGTTCTGTCCCCCTTGCCGTCCC 60
  ||||||||||||||||||||||||||||||||||||||||||||||||||||||||||||
Sbjct 252 CATTTTCAGACCTATGGAAACTACTTCCTGAAAACAACGTTCTGTCCCCCTTGCCGTCCC 311
Query 61  AAGCAATGGATGATTTGATGCTGTCCCCGGACGATATTGAACAATGGTTCACTGAAGACC 120
  ||||||||||||||||||||||||||||||||||||||||||||||||||||||||||||
Sbjct 312 AAGCAATGGATGATTTGATGCTGTCCCCGGACGATATTGAACAATGGTTCACTGAAGACC 371
Query 121  CAGGTCCAGATGAAGCTCCCAGAATGCCAGAGGCTGCTCCC**CGC**GTGGCCCCTGCACCAG 180
  |||||||||||||||||||||||||||||||||||||||||| |||||||||||||||||
Sbjct 372 CAGGTCCAGATGAAGCTCCCAGAATGCCAGAGGCTGCTCCC**CCC**GTGGCCCCTGCACCAG 431
Query 181  CAGCTCCTACACCGGCGGCCCCTGCACCAGCCCCCTCCTGGCCCCTGTCATCTTCTGTCC 240
  ||||||||||||||||||||||||||||||||||||||||||||||||||||||||||||
Sbjct 432 CAGCTCCTACACCGGCGGCCCCTGCACCAGCCCCCTCCTGGCCCCTGTCATCTTCTGTCC 491
Query 241  CTTCCCAGAAAACCTACCAGGGCAGCTACGGTTTCCGTCTGGGCTTCTTGCATTCTGGGA 300
  ||||||||||||||||||||||||||||||||||||||||||||||||||||||||||||
Sbjct 492 CTTCCCAGAAAACCTACCAGGGCAGCTACGGTTTCCGTCTGGGCTTCTTGCATTCTGGGA 551
Query 301  CAGCCAAGTCTGTGACTTGCACGTACTCCCCTGCCCTCAACAAGATGTTTTGCCAACTGG 360
  ||||||||||||||||||||||||||||||||||||||||||||||||||||||||||||
Sbjct 552 CAGCCAAGTCTGTGACTTGCACGTACTCCCCTGCCCTCAACAAGATGTTTTGCCAACTGG 611
Query 361  CCAAGACCTGCCCTGTGCAGCTGTGGGTTGATTCCACACCCCCGCCCGGCACCCGCGTCC 420
  ||||||||||||||||||||||||||||||||||||||||||||||||||||||||||||
Sbjct 612 CCAAGACCTGCCCTGTGCAGCTGTGGGTTGATTCCACACCCCCGCCCGGCACCCGCGTCC 671
Query 421  GCGCCATGGCCATCTACAAGCAGTCACAGCACATGACGGAGGTTGTGAGGCGCTGCCCCC 480
  ||||||||||||||||||||||||||||||||||||||||||||||||||||||||||||
Sbjct 672 GCGCCATGGCCATCTACAAGCAGTCACAGCACATGACGGAGGTTGTGAGGCGCTGCCCCC 731
Query 481  ACCATGAGCGCTGCTCAGATAGCGATGGTCTGGCCCCTCCTCAGCATCTTATCCGAGTGG 540
  ||||||||||||||||||||||||||||||||||||||||||||||||||||||||||||
Sbjct 732 ACCATGAGCGCTGCTCAGATAGCGATGGTCTGGCCCCTCCTCAGCATCTTATCCGAGTGG 791
Query 541  AAGGAAATTTGCGTGTGGAGTATTTGGATGA**C--A**AACACTTTTCGACATAGTGTGGTGG 598
  |||||||||||||||||||||||||||||||| ||||||||||||||||||||||||||
Sbjct 792 AAGGAAATTTGCGTGTGGAGTATTTGGATGA**CAGA**AACACTTTTCGACATAGTGTGGTGG 851
Query 599  TGCCCTATGAGCCGCCTGAGGTTGGCTCTGACTGTACCACCATCCACTACAACTACATGT 658
  ||||||||||||||||||||||||||||||||||||||||||||||||||||||||||||
Sbjct 852 TGCCCTATGAGCCGCCTGAGGTTGGCTCTGACTGTACCACCATCCACTACAACTACATGT 911
Query 659  GTAACAGTTCCTGCATGGGCGGCATGAACCGGAGGCCCATCCTCACCATCATCACACTGG 718
  ||||||||||||||||||||||||||||||||||||||||||||||||||||||||||||
Sbjct 912 GTAACAGTTCCTGCATGGGCGGCATGAACCGGAGGCCCATCCTCACCATCATCACACTGG 971
Query 719  AAGACTCCAGTGGTAATCTACTGGGACGGAACAGCTTTGAGGTGCGTGTTTGTGCCTGTC 778
  ||||||||||||||||||||||||||||||||||||||||||||||||||||||||||||
Sbjct 972 AAGACTCCAGTGGTAATCTACTGGGACGGAACAGCTTTGAGGTGCGTGTTTGTGCCTGTC 1031
Query 779  CTGGGAGAGACCGGCGCACAGAGGAAGAGAATCTCCGCAAGAAAGGGGAGCCTCACCACG 838
  ||||||||||||||||||||||||||||||||||||||||||||||||||||||||||||
Sbjct 1032 CTGGGAGAGACCGGCGCACAGAGGAAGAGAATCTCCGCAAGAAAGGGGAGCCTCACCACG 1091
Query 839  AGCTGCCCCCAGGGAGCACTAAGCGAGCACTGCCCAACAACAYCAGCTCCTCTCCCCAGC 898
  |||||||||||||||||||||||||||||||||||||||||| |||||||||||||||||
Sbjct 1092 AGCTGCCCCCAGGGAGCACTAAGCGAGCACTGCCCAACAACACCAGCTCCTCTCCCCAGC 1151

### Appendix C.2. List of Changes to the Data Bank Sequence

Codon 72: CCC changes to CGC: Prolin to Arginine a common polymorphism.

Codon 209 AGA: A and G deleted. DELETION causes FRAMESHIFT, truncated protein occurs DNA binding domain involved. This mutation is identical with the one described as p.R209fs*6 (Deletion-Frameshift) in the COSMIC (Catalogue of Somatic Mutations in Cancer) database. The mutated genotype is also mentioned in Reference [37].

Western blot detection of p53 protein in protein lysate of SCC-25 cells using the mouse monoclonal DO-7 antibody. Detroit 562 cell lysates, with a known gain of function mutation were used as a positive control.

SCC-25 cells contain very week, almost not visible band of p53 protein, while Detroit 562 cells synthesize strongly detectable protein. The p.R209fs*6 mutant protein product might be unstable and it degrades in the SCC-25 cells. As loading control: β-actin was used.
