# Supplementary material for: Nerve Growth Factor (NGF)—Receptor Survival Axis in Head and Neck Squamous Cell Carcinoma
Source: Int J Mol Sci. 2018 Jun 14;19(6):1771. doi: 10.3390/ijms19061771 (PMC6032238; doi:10.3390/ijms19061771)
Supplement: Supplementary file 1 [file ijms-19-01771-s001.pdf]

International Journal of Molecular Sciences

Supplementary Information

1. Supplementary Methods

1.1. Embedding

Tumor and UPPP samples were fixed in 4 % formaldehyde solution (SAV, Liquid Production GmbH, Flintsbach am Inn, Germany) overnight and were kept in PBS supplied with 1 g/L sodium azid until embedding, which was done using a Histos 5 (Histocom, Wr. Neudorf, Austria) paraffin embedding system, following the instructions of the manufacturer. After embedding, biopsies were sectioned and used for in situ hybridization and immunohistochemistry. Embedded specimens were serially sectioned at 5 µm thickness using a HM 355S microtome (Microm, Walldorf, Germany) and affixed onto Superfrost™ Plus slides (Menzel, Braunschweig, Germany). The mounted specimens were then dried overnight at room temperature, following which the slides were incubated at 60 °C for 1 hour to enable the sectioned specimens to adhere firmly onto the glass surface.

1.2. Immunohistochemistry

Immunohistochemistry was performed utilizing a Ventana Roche® Discovery Immunostainer (Mannheim, Germany), applying a DAB-MAP discovery research standard procedure or FISH procedure. Antigen retrieval was performed by epitope unmasking via a heat induction methodology performed while the sections were immersed in EDTA buffer (Cell Conditioning Solution CC1, Ventana 950-124).

Specimen affixed slides were incubated with appropriate primary antibodies at 37°C for 1 hour. The primary antibodies were detected with Discovery Universal Secondary Antibody (Ventana 760-4250) or by anti-mouse or anti-rabbit Alexa 488 or Alexa 594 conjugated secondary antibodies (Invitrogen, Eugene, Oregon, USA) incubating for 30 min in the Ventana Discovery immunostainer. Antibody detection was then attained employing the DAB-MAP Detection Kit (Ventana 760-124) utilizing a combinatorial approach involving the diaminobenzidine development method with copper enhancement followed by light counter staining with haematoxylin (Ventana 760-2021) for 4 minutes. The stained sections were then manually dehydrated using upgraded alcohol series, clarified with xylene and then mounted permanently with Entellan® (Merck, Darmstadt, Germany). The Alexa fluorochrome conjugated secondary antibody signals were detected after 5 minutes DAPI (Invitrogen) counterstaining on Vectashield mounted slides using fluorescent microscopy. The entire immunohistochemical staining reaction was benchmarked against appositive controls (e.g., cochlea, brain) that were supplemented to each experiment. Auxiliary negative controls were acquired by alternating the primary antibodies with reaction buffer or substituting them with isotype matching immunoglobulins. These auxiliary negative controls never yielded any immunostaining.

1.3. Riboprobe synthesis

Human NGF specific riboprobes were synthesized using the following primers: forward: CACACTGAGGTGCATAGCGT; reverse: TGATGACCGCTTGCTCCTGT. The DNA product was 389 base pairs long and was synthesized using Go-Taq Green Master Mix (Promega, Madison, WI, USA) and cDNA reverse transcribed from mRNA isolated from SCC-25 oral squamous cell carcinoma cell line. For the PCR reaction annealing temperature of 60 °C was used and the instructions of Promega were followed.

For production of template for riboprobes the T7 polymerase promoter sequence (5`-TAATACGACTCACTATAGGGAGA-3') was added to the reverse primer, and PCR product containing the T7 sequence before the reverse primer sequences was synthesized using the same conditions as above. 200 ng PCR products were Sanger sequenced by Microsynth (Vienna, Austria), using T7 promoter, and the identification and orientation of the sequence following the T7 promoter was controlled using NCBI Blast (NIH, Bethesda, MD, USA) nucleotide sequence alignment tool. The

antisense orientation of the sequence following the T7 promoter was confirmed. For control purpose T7-conjugated sense control riboprobe was used, which was showing only a minimal background reaction in cochlea and inner ear tissue. The DIG labelled riboprobes (antisense and control) were used in the same concentration for in situ hybridization. Digoxigenin (DIG) labelled riboprobes were synthesized and labelled using the T7 *in vitro* transcription kit of Roche Life Sciences (Cat. No. 11 175 025 910, Roche, Mannheim, Germany), and 1 µg of T7-containing template PCR products. The DIG labeling and the riboprobe concentration were determined using the DIG luminescent detection kit (Cat. Nr. 11 363 514 910, Roche) and CDP-star substrate (Roche) following the instruction of the manufacturer, Roche Life Sciences. The yield of riboprobe after in vitro transcription was by 600 ng/µl, altogether: 12 µg in 20 µl reaction mixture.

1.4. Sequence and annealing of the NGF antisense riboprobe

Annealed with Homo sapiens nerve growth factor (NGF), mRNA

```
Query  1    GTGGCGGTGGTCTTATCCCCAACCCACACGCTGACACTGTCACACACCGAGAATTCGCCC  60
            ||||||||||||||||||||||||||||||||||||||||||||||||||||||||||||
Sbjct  618  GTGGCGGTGGTCTTATCCCCAACCCACACGCTGACACTGTCACACACCGAGAATTCGCCC  559

Query  61   CTGTGGAAGATGGGATGGGATGATGACCGCTTGCTCCTGTGAGTCCTGTTGAAGGGGGCA  120
            ||||||||||||||||||||||||||||||||||||||||||||||||||||||||||||
Sbjct  558  CTGTGGAAGATGGGATGGGATGATGACCGCTTGCTCCTGTGAGTCCTGTTGAAGGGGGCA  499

Query  121  GCACCACCGACCTCGAAGTCCAGATCCTGAGTGTCTGCAGCTTCACGGGGAGGCTGGGTG  180
            ||||||||||||||||||||||||||||||||||||||||||||||||||||||||||||
Sbjct  498  GCACCACCGACCTCGAAGTCCAGATCCTGAGTGTCTGCAGCTTCACGGGGAGGCTGGGTG  439

Query  181  CTAAACAGCACACGGGGTGAACGGAGTCGCCGCTTTTTAAACAGCCTGGGGTCCACAGTA  240
            ||||||||||||||||||||||||||||||||||||||||||||||||||||||||||||
Sbjct  438  CTAAACAGCACACGGGGTGAACGGAGTCGCCGCTTTTTAAACAGCCTGGGGTCCACAGTA  379

Query  241  ATGTTGCGGGTCTGCCCCGCCACGCGTGCAGCTATCGCCGCTGCCGGGGCGCTGCGGGCT  300
            ||||||||||||||||||||||||||||||||||||||||||||||||||||||||||||
Sbjct  378  ATGTTGCGGGTCTGCCCCGCCACGCGTGCAGCTATCGCCGCTGCCGGGGCGCTGCGGGCT  319

Query  301  CTGCGAAGGGCAGTGTCAAGGGAATGCTGAAGTTTAGTCCAGTGGACTTGGGGGATGGTG  360
```

```
          |||||||||||||||||||||||||||||||||||||||||||| ||||||||||||||
Sbjct   318   CTGCGAAGGGCAGTGTCAAGGGAATGCTGAAGTTTAGTCCAGTGGGCTTGGGGGATGGTG
259

Query   361   TGTCCTGCAGGGACATTGCTCTCTGAGTGTGGTTCCGCCTGTATGCCGATCAGAAAAGCT
420
          ||||||||||||||||||||||||||||||||||||||||||||||||||||||||||||
Sbjct   258   TGTCCTGCAGGGACATTGCTCTCTGAGTGTGGTTCCGCCTGTATGCCGATCAGAAAAGCT
199

Query  421  GTGATCAGAGTGTAGAACAACATGGACATTACGCTATGCACCTCAGTGTGGCCAGG  476
            ||||||||||||||||||||||||||||||||||||||||||||||||||||||||
Sbjct  198  GTGATCAGAGTGTAGAACAACATGGACATTACGCTATGCACCTCAGTGTGGCCAGG  143
```

Annealed to nucleotides 143–618 in antisense orientation.

Table S1. Used antibodies.

Antibody	Host, clonality	Ab Dilution, Pre-treatment	Supplier Cat. No.
p75 NGF (p75NTR)	Rabbit monoclonal	1:160 CC1 standard	Abcam Cambridge, UK ab52987
p75 NGF (p75NTR)	Mouse monoclonal	1:1000 CC1 standard	Sigma, N5408
NTRK1 (12G8)	Rabbit monoclonal	1:400 CC1 standard	Cell Signalling, MS, USA
Pan - cytokeratin	Mouse monoclonal	Prediluted CC1 mild, Proteinase 3	Roche, Ventana 760-2595
Anti – DIG Rhodamin Fab Fragments	Sheep polyclonal	1:200	Roche Life Sciences 11207750910
Anti – DIG AP FAb Fragments	Sheep polyclonal	1:500	Roche Life Sciences 11093274910
Secondary antibody, anti – mouse Alexa 488	Donkey polyclonal	1 :200	Jackson Immunoresearch JAC715547003
Secondary antibody, anti – rabbit Alexa 594	Donkey polyclonal	1 :200	Life Technologies A21207

1.5. Intensity quantification

The TrkA and p75 antibodies reacted samples were scanned and photographed with Tissue Faxs (Tissue Gnostics Medical & Biotech Solutions, Vienna, Austria). Negative control was stained with isotype control immunoglobulins and did not contain any visible DAB reaction. A 2.5 × objective was used for the preview and a 20 x objective was utilized for the acquisition. As master channel for the focus Hematoxylin was used. The density quantification was done with the software Histo Quest (Tissue Gnostics; **Figure S1A,B**).

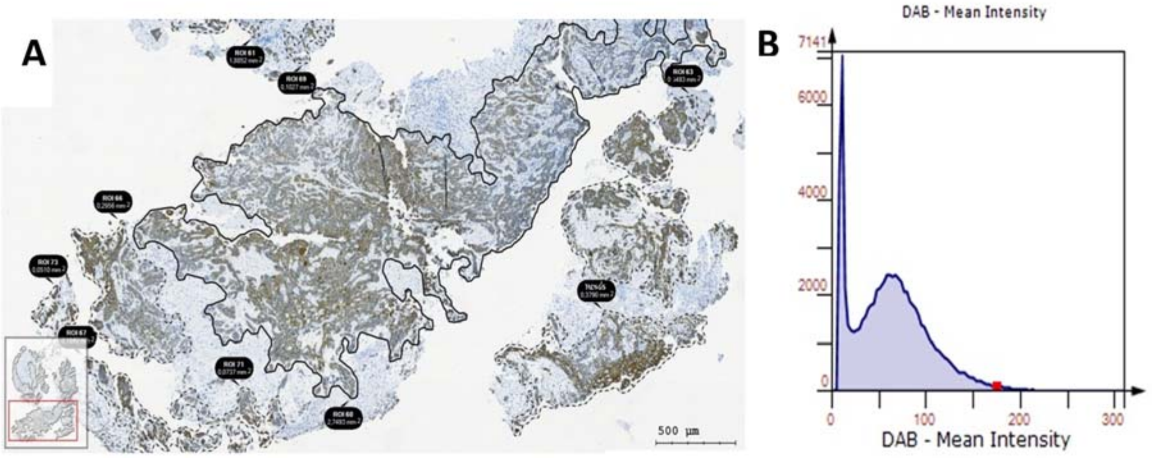

Figure S1. (**A**): Tissue recognition of HNSCC in Histoquest program; (**B**): Histogram (frequency diagram) of DAB mean intensity in recognized tumor cell nests.

Tumor cell nests were recognized automatically by tissue recognition algorithm of Histoquest based on their increased DAB and Hematoxylin.

The Histoquest used an algorithm to identify individual tumor cells in tumor cell nests based on their blue cell nuclei counterstained by hematoxylin (**Figure S2**). The default option was slightly modified by the user to avoid too fragmented cell nuclear recognition. The mean DAB grey value intensity was recognized for each and every identified tumor cells, and was averaged by the program for all samples. Finally, we received one representative intensity value per sample. An additional option was the determination of the % of cells identified with DAB—staining above the threshold of the negative control (**Figure S3**).

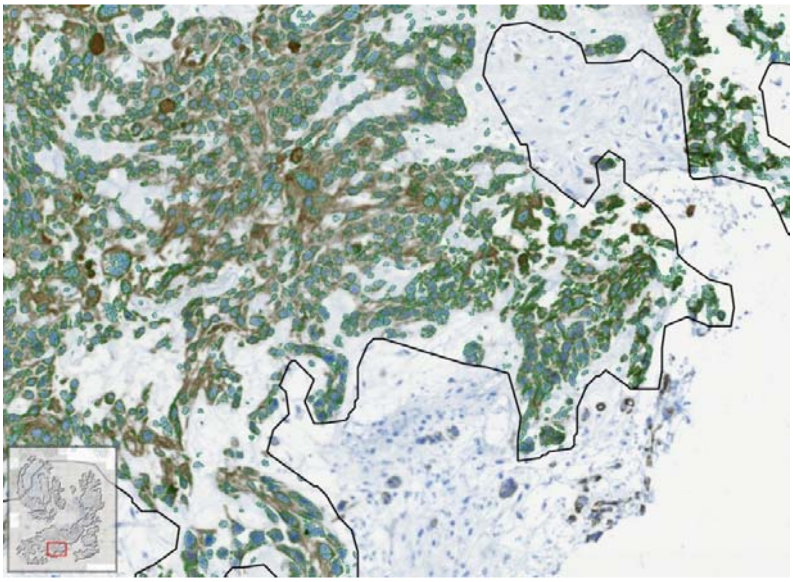

Figure S2. Identification of tumor cells in tumor cell nests.

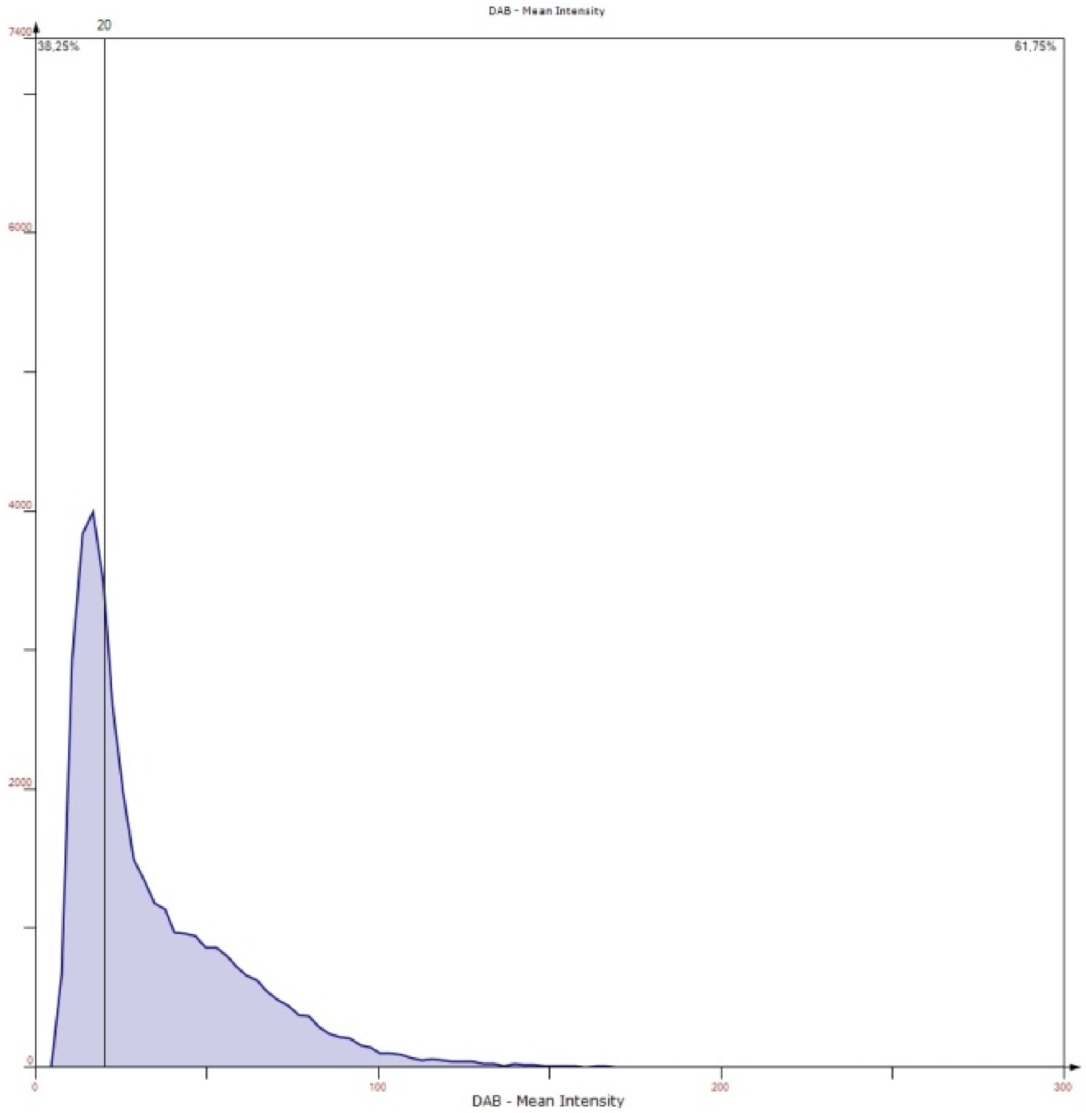

Figure S3. Histogram of epithelial layer immunostained with TrkA antibody. The vertical line at DAB mean intensity "20" represents the intensity value of the negative control. This histogram shows the 61.75% of cells are above the threshold (= are positive).
